# Characteristics of first-time fathers of advanced age: a Norwegian population-based study

**DOI:** 10.1186/1471-2393-13-29

**Published:** 2013-01-30

**Authors:** Anne Britt Vika Nilsen, Ulla Waldenström, Svein Rasmussen, Anna Hjelmstedt, Erica Schytt

**Affiliations:** 1 Department of Women’s and Children’s Health, Karolinska Institutet, Stockholm, Sweden; 2 Centre for Evidence Based Practice, Faculty of Health and Social Sciences, Bergen University College, Postboks 7030, 5020, Bergen, Norway; 3 Institute of Clinical Medicine, University of Bergen, Bergen, Norway; 4 Centre for Clinical Research Dalarna, Falun, Sweden

**Keywords:** Advanced paternal age, Postponing childbirth, Characteristics, First-time fathers

## Abstract

**Background:**

The modern phenomenon of delayed parenthood applies not only to women but also to men, but less is known about what characterises men who are expecting their first child at an advanced age. This study investigates the sociodemographic characteristics, health behaviour, health problems, social relationships and timing of pregnancy in older first-time fathers.

**Methods:**

A cross-sectional study was conducted of 14 832 men who were expecting their first child, based on data from the Norwegian Mother and Child Cohort Study (MoBa) carried out by the Norwegian Institute of Public Health. Data were collected in 2005–2008 by means of a questionnaire in gestational week 17–18 of their partner’s pregnancy, and from the Norwegian Medical Birth Register. The distribution of background variables was investigated across the age span of 25 years and above. Men of advanced age (35–39 years) and very advanced age (40 years or more) were compared with men aged 25–34 years by means of bivariate and multivariate logistic regression analyses.

**Results:**

The following factors were found to be associated with having the first child at an advanced or very advanced age: being unmarried or non-cohabitant, negative health behaviour (overweight, obesity, smoking, frequent alcohol intake), physical and mental health problems (lower back pain, cardiovascular diseases, high blood pressure, sleeping problems, previous depressive symptoms), few social contacts and dissatisfaction with partner relationship. There were mixed associations for socioeconomic status: several proxy measures of high socioeconomic status (e.g. income >65 000 €, self-employment) were associated with having the first child at an advanced or very advanced age, as were several other proxy measures of low socioeconomic status (e.g. unemployment, low level of education, immigrant background).The odds of the child being conceived after in vitro fertilisation were threefold in men aged 34–39 and fourfold from 40 years and above.

**Conclusions:**

Men who expect their first baby at an advanced or very advanced age constitute a socioeconomically heterogeneous group with more health problems and more risky health behaviour than younger men. Since older men often have their first child with a woman of advanced age, in whom similar characteristics have been reported, their combined risk of adverse perinatal outcomes needs further attention by clinicians and researchers.

## Background

The modern phenomenon of delayed parenthood applies not only to women but also to men [1-3], and men are often around three years older than women when having their first child. In Norway during the period 1975 to 2011, the mean age of first-time fathers increased from 26 to 31 years, and in first-time mothers from 23 to 28 years (http://www.ssb.no/fodte/tab-2012-04-11-04.html). The postponement of parenthood has been explained by factors such as rising female employment, expansion of university education, gender equity, partnership formation, delays in leaving the parental home, financial insecurity among young adults, family policies and effective contraception [4,5].

Research on obstetric and infant outcomes has mainly focused on the consequences of advanced maternal age [5,6] and less on the role of fathers. However, a review by Sartorius and Nieschlag [7] concluded that increasing paternal age was not only associated with fertility problems, but also with miscarriage, fetal death, very preterm birth, preeclampsia, caesarean section, and offspring problems such as birth defects, schizophrenia, autism, and cancer. Most of the studies in the review controlled for maternal age. Infertility and adverse obstetric and infant outcomes were explained by the association of increasing paternal age with declining androgen levels, deterioration in sperm quality and influences on the DNA integrity of the sperm. The authors also discussed possible effects of age-related cofactors, such as vascular diseases, accumulation of toxic substances and infections of the reproductive accessory glands.

When discussing consequences of advanced maternal and paternal age, it is often argued that adverse health outcomes should be weighed up against potential social advantages for the children, because the parents are more likely to have progressed in their careers and to have financial security [7,8]. While this may be true for most children of older parents, our recent findings in a study of the characteristics of older first-time mothers suggest that the picture is more complex [9]. In addition to having more age-related reproductive and physical health problems, women of advanced age constituted a heterogeneous group characterised by either socioeconomic prosperity or vulnerability. On the one hand, high maternal age was associated with high annual income; and on the other hand with a low level of education, single status, unemployment, an unsatisfactory partner relationship and an unplanned pregnancy.

In the present study, our aim was to investigate whether this pattern also applied to older first-time fathers. Thus, the aim was not to identify the effect of advanced paternal age on pregnancy, childbirth and infant outcomes, but only to describe the characteristics of older first-time fathers. Specifically, the aims were: 1) to give an overview of characteristics at different ages when having the first child, from the age of 25 years and above; and 2) to investigate associations between advanced and very advanced paternal age respectively, and sociodemographic background, health behaviour, physical and mental health problems, social relationships, and whether pregnancy was planned or a result of medically assisted reproduction.

## Methods

Selected data on first-time fathers were obtained from the Norwegian Mother and Child Cohort, a population-based cohort study conducted by the Norwegian Institute of Public Health. Participants were recruited from all over Norway from 1999–2008, and 38.5% of the invited women consented to participate. The cohort now includes 108 000 children, 90 700 mothers and 71 500 fathers. The method is described in previous publications [10,11]. In the period from 2000 to 2008, fathers-to-be from all over Norway were recruited to the study through a postal invitation, which was sent to the mothers-to-be in connection with the routine ultrasound examination at 17–18 weeks of gestation. The woman was asked to forward the invitation and a questionnaire to the father-to-be, and if he agreed to participate in the study, he returned his signed informed consent form and the completed questionnaire to the research team. For the present study, selected data about first-time fathers who filled in the questionnaire from April 2005–2008 (version V) was used, since the questionnaire version used during this period included full information relevant for our study. Data were also retrieved from the questionnaires filled in by their partners at the same time point. To assess the representativity of the study sample, we used data from the Norwegian Medical Birth Register, which includes information about all deliveries in Norway [12].

### Paternal age

Information about paternal age was obtained from the Norwegian Medical Birth Register and defined as age at the time of the baby’s birth. There is no consensus regarding the definition of ‘advanced’ paternal age. We chose age cut-offs based on five-year intervals, as in many other studies [13-17], and defined ‘advanced’ age as 35–39 years and ‘very advanced’ as ≥40 years. As a comparison group, we chose men aged 25–34 years, and excluded the youngest, who constitute a selected group with higher risk of negative exposures [14,18].

### Descriptive variables

Variables describing men’s characteristics were classified into four blocks, in accordance with the second aim of the study.

### Sociodemographics

Block 1 included the following sociodemographic characteristics: mother tongue (Norwegian vs other than Norwegian), ongoing or completed education (primary school, secondary school, higher education ≤4 years, higher education >4 years), employment (employed, self-employed, student, unemployed/disabled/rehabilitation), annual income (<200,000 NOK - ≥500,000 NOK) and civil status (married/cohabiting vs single).

### Health behaviour and health problems

Block 2 included health behaviour at the time of early pregnancy: smoking (no, yes daily, yes sometimes), alcohol usage (frequency and amount), physical activity (frequency), body mass index; physical health problems: migraine, headache, asthma, diabetes, cancer, cardiovascular disease, high blood pressure, abdominal pain, Crohn’s disease/ulcerative colitis, prolonged muscle pain, Mb Bechterew/rheumatoid arthritis, lower back pain, neck and shoulder pain, sexually transmitted diseases (chlamydia, genital herpes or warts, gonorrhoea); and mental health problems: sleeping problems, previous depressive symptoms, psychological distress. Questions about physical health problems and sleeping problems were phrased ‘*Do you have, or have you had any of the following illnesses or health problems?*’ followed by a list of symptoms. Previous depressive symptoms were measured by the Lifetime Major Depression Scale [19]. After the question *‘Have you ever experienced the following for a period of two weeks or more earlier in life?’* the respondent was asked to tick *yes* or *no* after the following statements: ‘1=Felt depressed, sad’, ‘2=Had problems with appetite or eaten too much’, ‘3=Been bothered by feeling weak or lack of energy’, ‘4=Really blamed yourself and felt worthless’, ‘5=Had problems with concentration or had problems making decisions’, and ‘6=Had at least three of the problems named above simultaneously’. Respondents who ticked *yes* on items 1 and 6 were classified as having previous depressive symptoms [20]. Current psychological distress was measured using a short form of the Symptom Checklist (SCL-5) [21,22]. The question ‘*Have you been bothered by any of the following feelings during the past two weeks?*’ was followed by the items: ‘feeling fearful’, ‘nervousness or shakiness inside’, ‘feeling hopeless about the future’, ‘feeling blue’, and ‘worrying too much about things’. Each item is scored on a 4-point scale (1=not bothered, 2=a little bothered, 3=quite bothered and 4=very bothered) with the total sum ranging from 5 to 20. Mean scores were calculated and a cut-off at ≥2 was defined as psychological distress [23].

### Present pregnancy

Block 3 included variables retrieved from the partner’s (woman’s) questionnaire and related to whether the present pregnancy was unplanned (Yes/No). The woman was asked if she had been treated for infertility in relation to the present pregnancy, and if so what type of medically assisted reproduction (MAR) treatment she had received: hormone treatment, intra-vaginal insemination or in vitro fertilisation (IVF) [24].

### Social relationships

Block 4 included the following variables related to social relationships: feeling lonely, having a support person other than partner, contacts with family and friends, and satisfaction with partner relationship. Satisfaction with partner relationship was measured using the five-item Relationship Satisfaction Scale [25], which is a shortened and modified version of the Marital Satisfaction Scale [26]. It includes the items: *‘My partner and I have problems in our relationship’*, ‘*I am very happy in my relationship*’, ‘*My partner is usually understanding’*, ‘*I am satisfied with my relationship with my partner*’ and ‘*We agree about how children should be raised*’. Each item is scored on a 6-point Likert scale with the end points ‘Completely agree’ and ‘Disagree completely’. The total sum ranges from 5 to 30. A mean score was computed for each individual, which was then dichotomised into dissatisfied (score <4) and satisfied (scores 4–6) [25]. In cases of a maximum of two missing values on either of the two five-item scales, answers were imputed; imputation was performed in 0.8% of the cases on the SCL-5 scale, and in 1.2% of the cases on the Relationship Satisfaction Scale [27].

### Statistical analyses

To assess the representativity of the study sample, we compared the men with all other first-time fathers in Norway in the same period, 2005–2008, using data from the Norwegian Medical Birth Register and the *χ*^2^-test for analysis. Figures were constructed showing the distribution of background characteristics by paternal age from 25 years to ≥45 years, and also the fathers’ age in relation to the age of the babies’ mothers. To investigate possible associations between ‘advanced’ and ‘very advanced’ paternal age respectively and all the descriptive variables, analyses were conducted in three steps. First, all the variables were tested one by one in bivariate analyses. Second, a multivariate logistic regression analysis of the statistically significant variables was conducted for each block of variables. Third, final multivariate logistic regression models were constructed, one for each age category, in which blocks 1 to 4 were entered one by one in a sequential order. Variables were left in the models if statistically significant (p<0.05) in one or both age categories. Only the final models are presented as crude and adjusted odds ratios (OR) with 95% confidence intervals (CI 95%). Internal missing values were between 0.0-3.0%, except for the alcohol variables (3.2-3.4%) and social relationship variables (0.6-1.7%). To retain cases with one or more missing values for categorical variables in the final model, a specific category ‘missing’ was constructed (not shown). Single imputations were made using Missing Value Analysis (MVA) and the Expectation Maximization (EM) algorithm method [28]. The remaining items on the scales were used as predictors for these imputations [27]. Collinearity for the final model was assessed using condition index.

The explained variance (Nagelkerke R^2^) of the two models was calculated for each block separately when entered into the models (one by one), and the cumulative explained variance was calculated when each block was added to the preceding blocks in the order described above.

The Statistical Package SPSS for Windows, version 19.0 (SPSS INC., Chicago, Illinois, USA), was used for the statistical analyses. The study was approved by the Regional Committee for Ethics in Medical Research and the Norwegian Data Inspectorate: (S-97045) and (2012/198 B).

## Results

A total of 127 231 pregnant women were asked to forward an invitation also to the baby’s father to join the Mother and Child Cohort study during the study period; 36 879 (29%) men consented to participate and received the questionnaire, and 33 944 (92%) actually responded. From this group, we excluded men who had at least one previous child (n=17 925) and the youngest first-time fathers of ≤24 years (n=1187) who were beyond the scope of this study, leaving a final sample of 14 832 first-time fathers.

Table 1 shows the characteristics of the sample compared with all first-time fathers in Norway during the study period. Men in the study sample differed from those in the Norwegian cohort of new fathers aged 25 years and above: men aged 35–39 were slightly overrepresented and men ≥40 years were slightly underrepresented. Additionally, more men in the study sample had a high level of education and fewer had a low level of education, or a mother tongue other than Norwegian.

**Table 1:** Characteristics of the study sample of first-time fathers (n = 14 832) compared with all first-time fathers in Norway (n = 211 762)

	**Study sample**		All first-time fathers in Norway*		
	**n**	**%**	**n**	**%**	p-value**
**Age groups**					<0.001
25-34 years	11 363	76.6	163 063	77.0	
35-39 years	2 693	18.2	34 291	16.2	
≥40 years	776	5.2	14 408	6.8	
**Mother tongue other than Norwegian**	1 016	6.9	46 293	21.9	<0.001
Education					<0.001
Primary school	288	2.0	29 511	13.9	
Secondary school	4 862	33.7	87 850	41.5	
Higher education ≤4 years	4 301	29.8	53 193	25.1	
Higher education >4 years	4 984	34.5	27 635	13.1	
Unknown	397	2.7	13 573	6.4	

*Data from the Norwegian Medical Birth Register.

**P-value for differences between the first-time fathers in the study sample and first-time fathers in Norway 2005-2008.

**Figure 1:**
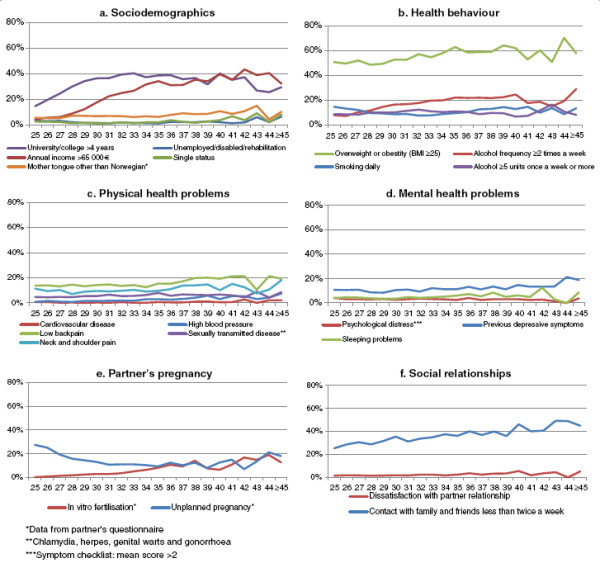
a-f. Background variables in relation to age when becoming first-time fathers.

**Figure 2:**
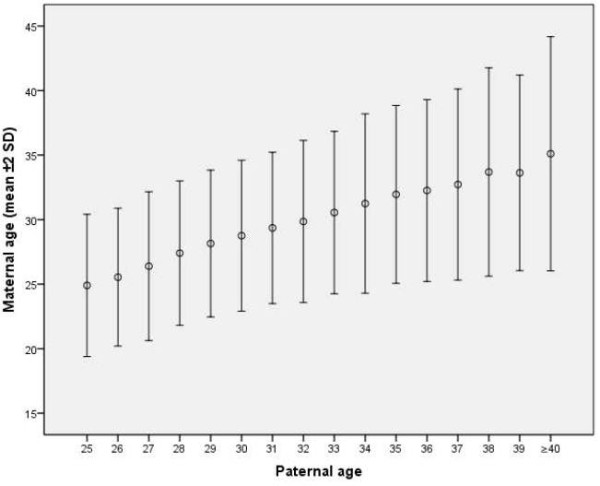
First-time father's age in relation to the age of his baby's mother (mean, ±2SD) (n=14 832).

Figure 1a shows that, although university/college education (>4 years) and high annual income increased by paternal age, these outcomes peaked in men aged 33 and 42 years respectively, and were slightly less common in the oldest men. Unemployment, single status, and native languages other than Norwegian were somewhat more common among the oldest. Being overweight or obese seemed to increase with age (Figure 1b); physical and mental health problems increased from the age of about 35 years (Figure 1c, d); and, compared with the youngest group, the partner’s pregnancy was more often planned or a result of IVF (Figure 1e). Social contacts with family, other than their partner, and friends seemed to decline gradually with age, but satisfaction with the partner relationship was about the same in all age groups (Figure 1f). Figure 2 shows the mean maternal age in relation to the age of first-time fathers. The mean age in the mothers increased with paternal age: the mean was 32.6 years and the median 33 for the partners of men of advanced age (35–39 years); and the mean was 35.1 years and the median 36 for the partners of men of very advanced age (≥40 years). Having a partner younger than themselves was more common for fathers of advanced and very advanced age than for younger men.

Table 2 shows the crude and adjusted odds ratios for men of advanced age and very advanced age compared with the comparison group of 25–34 year-olds regarding sociodemographic characteristics (Block 1), health behaviour and health problems (Block 2), planning of pregnancy (Block 3) and social relationship variables (Block 4).

**Table 2:** Associations between paternal age and sociodemographic characteristics (Block 1), health behaviour, physical and mental health problems (Block 2) partner's present pregnancy (Block 3) and social relationships (Block 4) in expectant first-time fathers (35-39 years and ≥40 years) compared with a reference group aged 25-34 years

	**25-34 years**		**35-39 years**						**≥40 years**					
	**n=11 363**		**n=2 693**						**n=776**					
	**n**	**%**	**n**	**%**	**Crude OR**	**(95**% **CI)**	**OR**^**a**^	**(95**% **CI)**	**n**	**%**	**Crude OR**	**(95% CI)**	**OR**^**a**^	**(95% CI)**
*Sociodemographics (Block 1)*														
Mother tongue other than Norwegian^bc^	733	6.5	205	7.6	1.19	(1.02-1.40)	1.26	(1.06-1.50)	78	10.1	1.62	(1.27-2.07)	1.40	(1.07-1.84)
*Education*														
Primary school	191	1.7	62	2.3	1.48	(1.10-1.99)	1.55	(1.13-2.13)	35	4.5	2.75	(1.88-4.03)	2.67	(1.74-4.10)
Secondary school	3 780	33.3	830	30.8	ref		ref		252	32.5	ref		ref	
Higher education ≤4 years	3 368	29.6	731	27.1	0.99	(0.89-1.10)	0.96	(0.86-1.09)	202	26.0	0.90	(0.74-1.09)	0.90	(0.73-1.11)
Higher education >4 years	3 720	32.7	1 000	37.1	1.22	(1.10-1.36)	1.03	(0.91-1.16)	264	34.0	1.06	(0.89-1.27)	0.83	(0.68-1.03)
*Employment*														
*Employed*	8 968	78.9	2 192	81.4	ref		ref		592	76.3	ref		ref	
*Self-employed*	1 066	9.4	328	12.2	1.26	(1.10-1.44)	1.42	(1.23-1.63)	129	16.6	1.83	(1.50-2.24)	1.92	(1.54-2.39)
*Student*	804	7.1	54	2.0	0.27	(0.21-0.36)	0.51	(0.37-0.68)	11	1.4	0.21	(0.11-0.38)	0.39	(0.21-0.73)
Unemployed/disabled/rehabilitation	216	1.9	50	1.9	0.95	(0.69-1.29)	1.47	(1.04-2.08)	25	3.2	1.75	(1.15-2.67)	2.02	(1.23-3.33)
Annual income NOK (Norwegian krone)														
<200 000	1 271	11.2	114	4.2	0.46	(0.38-0.57)	0.48	(0.38-0.60)	39	5.0	0.61	(0.43-0.86)	0.50	(0.33-0.74)
200-299 999	1 765	15.5	256	9.5	0.75	(0.64-0.87)	0.69	(0.59-0.81)	82	10.6	0.92	(0.71-1.20)	0.69	(0.52-0.92)
300-399 999	3 995	35.2	775	28.8	ref		ref		201	25.9	ref		ref	
400-499 999	2 262	19.9	633	23.5	1.44	(1.28-1.62)	1.42	(1.26-1.60)	155	20.0	1.36	(1.10-1.69)	1.40	(1.12-1.76)
≥500 000	1 916	16.9	887	32.9	2.39	(2.14-2.67)	2.43	(2.16-2.73)	290	37.4	3.01	(2.49-3.63)	3.29	(2.68-4.04)
Civil Status														
Single status^c^	182	1.6	66	2.5	1.45	(1.09-1.94)	1.90	(1.39-2.59)	44	5.7	3.16	(2.23-4.48)	3.58	(2.42-5.31)
*Health behaviour, physical and mental health problems(Block 2)*														
Body Mass Index (kg/m^2^)														
Normal (18.5-24.9)	5 319	46.8	1 050	39.0	ref		ref		322	41.5	ref		ref	
Overweight or obesity (≥25)	5 967	52.5	1 629	60.5	1.38	(1.27-1.51)	1.42	(1.29-1.55)	452	58.2	1.25	(1.08-1.45)	1.28	(1.09-1.50)
Underweight (<18.5)	28	0.2	4	0.1	0.72	(0.25-2.07)	0.84	(0.27-2.54	1	0.1	0.59	(0.08-4.35)	0.47	(0.05-4.19)
Smoking^c^														
Yes, sometimes	1 137	10.0	226	8.4	0.89	(0.76-1.03)	0.99	(0.85-1.17)	56	7.2	0.84	(0.63-1.12)	0.92	(0.69-1.24)
Yes, daily	1 075	9.5	302	11.2	1.26	(1.10-1.45)	1.41	(1.21-1.65)	98	12.6	1.52	(1.12-1.91)	1.46	(1.13-1.89)
Alcohol (frequency)														
Less than once a month or never	2 535	22.3	523	19.4	0.68	(0.60-0.77)	0.74	(0.65-0.84)	161	20.7	0.57	(0.47-0.70)	0.58	(0.46-0.72)
Once a week/month	6 803	59.9	1 487	55.2	ref		ref		412	53.1	ref		ref	
2-3 times a week	1 482	13.0	489	18.2	1.74	(1.54-1.96)	1.57	(1.39-1.79)	131	16.9	1.84	(1.48-2.27)	1.63	(1.30-2.04)
4-7 times a week	187	1.6	101	3.8	2.84	(2.20-3.67)	2.46	(1.88-3.20	40	52	4.34	(2.97-6.34)	3.59	(2.40-5.36)
Alcohol (≥5 units^e^ when consuming)														
Never or do not drink alcohol	1 800	15.8	525	19.5	1.17	(1.04-1.32)	1.51	(1.32-1.72)	210	27.1	1.63	(1.39-1.96)	2.25	(1.83-2.77)
Less than once per month	4 447	39.1	1 107	41.1	ref		ref		318	41.0	ref		ref	
1-3 times per month	3 595	31.6	708	26.3	0.79	(0.71-0.88)	0.67	(0.60-0.75)	147	18.9	0.57	(0.47-0.70)	0.44	(0.36-0.55)
Once or several times per week	1 157	10.2	267	9.9	0.93	(0.80-1.08)	0.64	(0.54-0.76)	69	8.9	0.83	(0.64-1.09)	0.50	(0.37-0.68)
Physical health problems^c^														
Cardiovascular disease	33	0.3	17	0.6	2.18	(1.21-3.92)	2.18	(1.16-4.09)	10	1.3	4.48	(2.20-9.13)	3.94	(1.79-8.66)
High blood pressure	179	1.6	90	3.3	2.16	(1.67-2.79)	1.69	(1.29-2.22)	39	5.0	3.31	(2.32-4.71)	2.60	(1.76-3.83)
Neck and shoulder pain	1 063	9.4	321	11.9	1.31	(1.15-1.50)	1.20	(1.03-1.39)	103	13.3	1.48	(1.19-1.84)	1.09	(0.85-1.40)
Low back pain	1 571	13.8	453	16.8	1.26	(1.12-1.41)	1.21	(1.07-1.37)	150	19.3	1.49	(1.24-1.80)	1.45	(1.18-1.79)
Mb Bechterew	63	0.6	16	0.6	1.07	(0.62-1.86)	0.85	(0.48-1.52)	13	1.7	3.06	(1.67-5.58)	2.16	(1.11-4.21)
Sexually transmitted diseases^d^	611	5.4	182	6.8	1.28	(1.07-1.51)	1.26	(1.05-1.51)	52	6.7	1.26	(0.94-1.69)	1.32	(0.96-1.81)
Mental health problems^c^														
Sleeping problems	476	4.2	174	6.5	1.58	(1.32-1.89)	1.45	(1.19-1.77)	52	6.7	1.64	(1.22-2.21)	1.20	(0.86-1.69)
Previous depressive symptoms	1 161	10.2	323	12.0	1.20	(1.05-1.37)	1.22	(1.05-1.41)	120	15.5	1.61	(1.31-1.97)	1.44	(1.14-1.82)
*Present pregnancy*^*bc*^*(Block 3)*														
Medically assisted reproduction														
Hormone treatment	302	2.7	106	3.9	1.63	(1.30-2.04)	1.43	(1.13-1.81)	32	4.1	1.75	(1.20-2.54)	1.70	(1.16-2.52)
Insemination	21	0.2	14	0.5	3.09	(1.57-6.09)	2.75	(1.34-5.63)	3	0.4	2.36	(0.70-7.92)	2.18	(0.60-7.90)
In vitro fertilisation	348	3.1	267	9.9	3.56	(3.01-4.20)	3.12	(2.62-3.72)	93	12.0	4.41	(3.46-5.62)	4.13	(3.17-5.39)
Unplanned pregnancy	1 695	14.9	290	10.8	0.69	(0.60-0.78)	0.79	(0.68-0.91)	111	14.3	0.96	(0.78-1.18)	1.04	(0.82-1.31)
*Social relationships (Block 4)*														
Contact with family and friends														
More than twice a week	7 574	66.7	1 645	61.1	ref		ref		426	54.9	ref		ref	
Twice a week or less	3 659	32.2	1 016	37.7	1.28	(1.17-1.40)	1.22	(1.11-1.34)	343	44.2	1.67	(1.44-1.93)	1.48	(1.26-1.73)
No other support persons than partner	803	7.1	234	8.7	1.25	(1.07-1.45)	1.14	(0.97-1.34)	102	13.1	1.98	(1.59-2.47)	1.55	(1.22-1.98)
Partner relationship														
Satisfaction with partner relationship	11 044	97.2	2 593	96.3	ref		ref		739	95.2	ref		ref	
Dissatisfaction with partner relationship	211	1.9	79	2.9	1.59	(1.23-2.07)	1.48	(1.11-1.96)	31	4.0	2.20	(1.50-3.22)	1.52	(0.99-2.36)

Values shown for variables remaining in the final model only (n=14 832, % presented by column).

^a^Odds ratio adjusted for all other variables in the model.

^b^Data from the partner's questionnaire.

^c^Reference: Men unexposed to the variable studied.

^d^Chlamydia, genital herpes, genital wart, gonorrhoea.

^e^1.5 cl. pure alcohol.

All sociodemographic variables differed from the comparison group. Unemployment, a low level of education and an immigrant background were features associated with having the first baby at an advanced or very advanced age, but affected only a few individuals. In contrast, the older men were more likely to have a high income (≥500 000 NOK (Norwegian krone) ≈65 000 €) and to be self-employed. The odds were nearly twofold and fourfold respectively that men of advanced and very advanced age would be unmarried or non-cohabiting.

Health behaviour, physical and mental health problems also differed with age. The older men were more likely to be overweight or obese, to be smokers and to consume alcohol more frequently than the comparison group; however, they were less likely to be heavy consumers. Physical health problems, such as low back pain, were more common in older fathers, and most common in those of advanced age. Few were affected by cardiovascular disease and high blood pressure, but the odds were higher for the oldest groups compared with the youngest. The mental health problems associated with high paternal age were sleeping problems (advanced age), and previous depressive symptoms (advanced and very advanced age), but not ongoing psychological distress.

An unplanned pregnancy was as common in the oldest group as in the youngest, although less likely in men aged 35–39 years. The odds of the baby having been conceived after medically assisted reproduction were threefold in men aged 34–39 and fourfold from 40 years and above, compared with the youngest group.

Finally, limited contact with family and friends, and lack of support from others apart from their partner, was more common for the oldest men. Very few reported a poor partner relationship, but the relationship seemed most problematic for men aged 35–39.

The following variables were not associated with paternal age in either of the groups in the final model: physical activity, asthma, migraine, headache, diabetes, cancer, stomach-ache, Crohn’s disease/ulcerative colitis, prolonged muscle pain, psychological distress and feeling lonely.

Table 3 shows that the models explained 12.7% (35–39 years) and 17.5% (≥40 years) of the variance, respectively. In the older age groups, most of the variance, 6.8% and 7.9% respectively, was explained by sociodemographic factors.

**Table 3:** **Explained variance (R2**% **by Nagelkerke) of having the first baby at an*advanced*and*very advanced*paternal age; 35-39 years vs ≥40 years, compared with men aged 25-34 years in four blocks of exposure variables**^**a**^

	**Explained variance of each block**		**Cumulative explained variance**	
	**35-39 yrs**	**≥40 yrs**	**35-39 yrs**	**≥40 yrs**
**Block**^**b**^	**R**^2^	**R**^2^	**R**^2^	**R**^2^
Sociodemographics	6.6	7.8	6.6	7.8
Health behaviour, physical and mental problems	4.0	6.8	10.5	14.3
Planning of pregnancy	2.7	2.7	12.5	16.5
Social relationships	0.5	1.7	12.8	17.4

^a^Blocks entered either as first block (Explained variance of each block) or in sequential order (Cumulative explained variance).

^b^Variables in each block as in Table 2.

## Discussion

This study reveals that men who become fathers for the first time at an advanced (35–39 years) or very advanced age (≥40 years) in Norway seem to constitute a heterogeneous group from a socioeconomic perspective: high-income earners were overrepresented, as well as men with a low level of education and men who were unemployed. It was also more common for the older men to be single or have less favourable social relationships, and they were at higher risk of negative health behaviour and health problems. Nevertheless, the vast majority lived up to the conditions set by many men when asked about the appropriate time for having the first baby; i.e. one should have a completed education, a permanent job and a stable financial situation, and be in a stable relationship [29,30]. Thus, the overall picture was very similar to the one reported in our previous study of first-time mothers [9].

As men do not face the same biological age limit for having children, more men than women have children at an advanced or very advanced age. Available data suggest that about 10% of fathers in the Nordic countries, Australia, England, Wales, and France are in their 40s, and a smaller percentage have children after 50 [5]. In the present population-based sample, 5.2% of the men were 40 years and above, compared with 6.8% in the national population of first-time fathers.

The medical risks associated with advanced paternal age have not gained the same level of attention as those related to advanced maternal age. This might be explained by the fact that men are able to become fathers later in life than women, and there has been less research into male factors than into those relating to females. The potential negative effect of advanced male age on reproductive outcome has been related to deterioration in sperm quality, but the specific age at which problems may occur cannot be easily defined. Studies of infertility are inconclusive; while one review has concluded that paternal age above the late 30s is a risk factor [31], another [7] specified that male age was a risk factor at least in couples where men are older than 40 and women are at least 35 years old. The present study only includes men where conception has been successful, and the issue of sperm quality in this context is relevant only in relation to its influence on obstetric and infant outcomes; however, many of the couples had experienced fertility problems.

The most important risk associated with advanced paternal age is related to having a baby with a woman of advanced age [5,6]. In first-time mothers, a range of obstetric and infant complications, such as caesarean section, preterm birth, and perinatal mortality increase with age, particularly from the mid-30s [5,32]. In our study, the median age of the partners of men of advanced and very advanced age was 33 and 36 years respectively. Thus, about 50% of these men had a partner who was at risk of age-related complications. Little is known about the combined effect of expecting the first child at an advanced male and advanced female age. In their recent review of the consequences of postponement of parenthood, Schmidt et al. [5,33] concluded that ‘as women in general have partners who are several years older than themselves, it is important to focus more on the combined effect of advanced female and advanced male age on reproductive outcomes in the future.’

We are not aware of any negative obstetric complications associated with the higher prevalence of low education, unemployment and single status, among first-time fathers of very advanced age. These characteristics may be harder to accept at the age of 40 and above than earlier in life, and may have a negative influence on the relationship between the new parents and the social context in which the baby is born. These men do not fit into the general picture of men who postpone pregnancy in order to obtain a more stable start for the newborn baby. The small group of socioeconomically disadvantaged men may have a more challenging transition into fatherhood, finding it difficult to establish optimal conditions for developing contact and attachment with the baby [34]. A qualitative study showed that working-class fathers were less likely to be involved in childrearing than middle-class fathers, and that they more easily adopted the traditional gender role as breadwinner [35].

In addition to the independent effect of advanced male age on obstetric and neonatal outcomes [7], male age is associated with a lower chance of achieving a live birth by IVF/ICSI treatment [36]. The older men in our study were at higher risk than younger men of being overweight or obese, of having high blood pressure, and of practising risky behaviour such as smoking and frequent alcohol consumption. Some of these factors may affect reproductive outcomes. Smoking, for example, negatively affects sperm production, motility and morphology, and is associated with an increased risk of DNA damage [37,38]. Obesity may have an indirect effect by increasing the risk of cardiovascular disease, diabetes, and some cancers [39], in addition to a direct effect on time to pregnancy (TTP) [33,40] especially in cases where also the woman is obese. Furthermore, the home environment in which the child will be brought up is influenced by the parents’ health behaviour [41].

The strength of this study is the large sample of first-time fathers, and data on fathers-to-be are seldom available from national statistics [1]. The low response rate may be a consequence of the recruitment process. Non-responding and less advantaged women [9], or women who did not have any contact with the baby’s father, would probably not assist in the recruitment by forwarding the invitation and questionnaire. Consequently the most vulnerable fathers-to-be, as well as couples, are not included in this study and the negative outcomes may therefore be underestimations.

## Conclusion and clinical implications

The men who became fathers for the first time at an advanced age or very advanced age constituted a socioeconomically heterogeneous group, where the vast majority had a stable financial and social situation, and a minority was characterised by a low level of education, unemployment or single status. Health problems and risky health behaviour were more common than in younger men. Overall, the characteristics were very similar to those reported in older mothers-to-be. Our findings may modify a relatively common view that older first-time fathers constitute a homogeneous group of well-established and resourceful individuals. This information may help clinicians who care for expectant and new parents to provide more individualised care and support.

The higher rates of health problems and risky health behaviours in the men of advanced and very advanced age, and the fact that these men often had their first child with a woman of advanced age, suggest that the risk of adverse obstetric and perinatal outcomes would increase. Although it is beyond the scope of this study to draw conclusions about the effects of increased paternal age, our findings highlight characteristics of older first-time fathers that should be further investigated, the most important being the combined effect of advanced paternal and maternal age. Even if specific knowledge about the combined effects is limited, there is sufficient evidence about the adverse negative effects of advanced maternal age on fertility and perinatal outcomes, and also about the negative effects of advanced paternal age on fertility and some infant outcomes, in order to support public health interventions focusing on information to young women and men, maybe already in school. There is, for example, a widespread misapprehension that assisted reproductive technology (ART) is a simple and effective solution to infertility problems. A Swedish study showed that male students were too optimistic regarding age-related female fecundity, and they also overestimated the chances of having a child through IVF treatment [30].

We therefore advocate that young women and men should be given more information about fecundity and the medical risks associated with postponing childbirth. Furthermore, we welcome investigations about effective incentives, such as facilitation of parental leave and improved financial conditions, for couples to conceive a few years earlier than is usual in modern societies of today.

## Competing interests

The authors declare that they have no competing interests.

## Authors' contributions

ABVN contributed to the planning of the study, and also analysed the data, contributed to the interpretation of findings and wrote the first draft of the manuscript. UW was the principal investigator and contributed with the idea, as well as to the interpretation of the results and the writing of the manuscript. AH contributed to the interpretation of results and by commenting on the manuscript. SR contributed in the analyses, and commented on the manuscript. ES contributed to the planning of the study, to the data analyses, to the interpretation of the results and to the writing of the manuscript. All of the authors have approved the final version of the manuscript.

## Pre-publication history

The pre-publication history for this paper can be accessed here:

http://www.biomedcentral.com/1471-2393/13/29/prepub
